# Predictors of anti-convulsant treatment failure in children presenting with malaria and prolonged seizures in Kampala, Uganda

**DOI:** 10.1186/1475-2875-8-145

**Published:** 2009-06-29

**Authors:** Arthur Mpimbaza, Sarah G Staedke, Grace Ndeezi, Justus Byarugaba, Philip J Rosenthal

**Affiliations:** 1 Department of Paediatrics and Child Health, Faculty of Medicine, Makerere University, Kampala, Uganda; 2 London School of Hygiene and Tropical Medicine, London, UK; 3 Department of Medicine, San Francisco General Hospital, University of California, San Francisco, California, USA

## Abstract

**Background:**

In endemic areas, falciparum malaria remains the leading cause of seizures in children presenting to emergency departments. In addition, seizures in malaria have been shown to increase morbidity and mortality in these patients. The management of seizures in malaria is sometimes complicated by the refractory nature of these seizures to readily available anti-convulsants. The objective of this study was to determine predictors of anti-convulsant treatment failure and seizure recurrence after initial control among children with malaria.

**Methods:**

In a previous study, the efficacy and safety of buccal midazolam was compared to that of rectal diazepam in the treatment of prolonged seizures in children aged three months to 12 years in Kampala, Uganda. For this study, predictive models were used to determine risk factors for anti-convulsant treatment failure and seizure recurrence among the 221 of these children with malaria.

**Results:**

Using predictive models, focal seizures (OR 3.21; 95% CI 1.42–7.25, p = 0.005), cerebral malaria (OR 2.43; 95% CI 1.20–4.91, p = 0.01) and a blood sugar ≥200 mg/dl at presentation (OR 2.84; 95% CI 1.11–7.20, p = 0.02) were independent predictors of treatment failure (seizure persistence beyond 10 minutes or recurrence within one hour of treatment). Predictors of seizure recurrence included: 1) cerebral malaria (HR 3.32; 95% CI 1.94–5.66, p < 0.001), 2) presenting with multiple seizures (HR 2.45; 95% CI 1.42–4.23, p = 0.001), 3) focal seizures (HR 2.86; 95% CI 1.49–5.49, p = 0.002), 4) recent use of diazepam (HR 2.43; 95% CI 1.19–4.95, p = 0.01) and 5) initial control of the seizure with diazepam (HR 1.96; 95% CI 1.16–3.33, p = 0.01).

**Conclusion:**

Specific predictors, including cerebral malaria, can identify patients with malaria at risk of anti-convulsant treatment failure and seizure recurrence.

## Background

In endemic areas, falciparum malaria remains the leading cause of seizures in children presenting to emergency departments [1-4]. In addition, seizures are the most common neurological complication of severe malaria, often resulting in hospital admission, and seizures have been shown to increase morbidity and mortality in these patients [1,4,5]. The management of seizures in malaria is complicated, as they may be refractory to readily available anti-convulsants and there is concern for drug induced respiratory depression [6].

Uncontrolled seizures in malaria can damage the brain by aggravating hypoxia, hypoglycaemia and intracranial hypertension, and in children with cerebral malaria they have been shown to increase the risks of neurological complications, cognitive impairment and death [5,7-10]. In addition, children with malaria and complicated seizures are at a significantly higher risk of developing epilepsy as compared to children without this complication [11].

Diazepam administered intravenously or rectally is the recommended first-line treatment for seizures in children with malaria [6,12]. However, use of diazepam is characterized by high failure rates, particularly with the rectal route of administration [13]. Even when seizures are controlled, relapse rates are high, probably due to the short duration of action of diazepam in the brain [6,13]. This limitation leads to repeated administration of diazepam, largely in settings where alternative safe and effective anti-convulsants are not available [14]. The key danger with repeated dosing of diazepam is respiratory depression [13,15], an uncommon occurrence with appropriate dosing of the drug [16]. Furthermore, the use of phenobarbital, a readily available second-line agent, in addition to diazepam may increase the risk of respiratory depression [17]. A good therapeutic alternative to diazepam is midazolam, which unlike diazepam is water-soluble and can be administered through the buccal and nasal routes [6,18,19]. Midazolam has been shown to be at least as effective and safe as diazepam in the management of seizures in children in Europe and in Africa [4,20-23].

Identification of patients at risk of failing anti-convulsant treatment or experiencing recurrences of seizures after initial control is a practical step toward improving the care of children with malaria complicated by seizures. If identified early these children could be provided with optimal anti-convulsants to control seizures and minimize the risk of respiratory depression. In this study, risk factors for anti-convulsant treatment failure and seizure recurrence among Ugandan children with malaria and prolonged seizures were identified.

## Methods

### Study design

Results of a randomized trial to compare the efficacy and safety of buccal midazolam versus rectal diazepam in children with persistent seizures were reported previously [4]. The study was conducted between November 2005 and June 2006 in the Acute Care Unit (ACU), the paediatric emergency unit of Mulago Hospital, the national referral hospital in Kampala, Uganda. Patients were enrolled if they fulfilled the following criteria: (1) three months to 12 years of age, (2) no documented evidence of having received IV diazepam or IV phenobarbital within 24 hours prior to presentation, (3) documented seizure persisting for more than five minutes at the time of administration of study drug, and (4) provision of informed consent to continue participation in the study. In view of practical limitations, informed consent was waived upon emergency presentation. Written consent to continue participation in the study was subsequently sought from parents or legal guardians as soon as was practically possible after initial evaluation and treatment.

Patients were randomly assigned to one of two treatment arms: rectal diazepam and buccal placebo or rectal placebo and buccal midazolam. Parenteral preparations of both diazepam (Roche, France) and midazolam (Roche, France) were prepackaged by a pharmacist in two boxes that corresponded to the two treatment arms. Boxes were stored at 5–10°C and emptied and refilled on a weekly basis. Diazepam and placebo were packaged in 2 ml glass syringes and buccal midazolam and placebo were packaged in 2 ml plastic syringes. Both re-packaged midazolam and diazepam are stable under these conditions for up to one month [24-26]. Both drugs were administered at approximately 0.5 mg/kg (2.5 mg for age 3 to11 months; 5 mg for age 1 to 4 years; 7.5 mg for age 5 to 9 years; 10 mg for age10 to12 years). The primary study outcome was cessation of visible seizure activity within 10 minutes of receiving study medications without recurrence in the subsequent one hour. If the seizure persisted beyond 10 minutes or recurred within one hour after initial control, the child was categorized as a treatment failure. Secondary outcome measures included: 1) proportion with cessation of convulsions within ten minutes, 2) proportion with seizure recurrence in subsequent hour and within 24 hours after initial control and 3) time to seizure recurrence. Any child presenting with fever, with asexual forms of *Plasmodium falciparum *detected in the blood, and without another illness explaining the clinical state was considered to have malaria. Cerebral malaria was defined as a child unable to localize painful stimuli with persistence of coma (Blantyre coma score ≤ 2) for more than an hour after control of the seizure, with peripheral parasitaemia and after the exclusion of other causes of encephalopathy, in particular central nervous system infections and hypoglycaemia [27]. Seizures were classified as focal, general, or focal with secondary generalization [28].

The Uganda National Council for Science and Technology and the institutional review boards of Makerere University, Kampala and the University of California, San Francisco approved the study.

### Data analysis

Statistical analysis was done using Stata 10.0 (Stata Corp, College Station, TX, USA). Multivariate analysis using logistic regression was used to identify predictors of treatment failure. Associations between potential predictors for treatment failure were assessed using cross-tabulations with chi-squared tests. The final model was selected using the Stata 10.0 backward selection procedure set at p < 0.20 and it included: 1) duration of seizures prior to receiving treatment, 2) form of disease (cerebral malaria vs. other forms of severe malaria), 3) seizure type, 4) age and 5) blood sugar level. For survival analysis, differences in survival functions between potential explanatory predictors of seizure recurrence were initially compared using Kaplan Meier curves and the log rank test. The Cox proportional hazard model was applied to assess the independent roles of potential predictors of seizure recurrence. Covariates with a significant log rank test (p ≤ 0.004) were included in the final model. Anti-convulsant treatment received to terminate the seizure was included in both the logistic regression model and Cox model to determine any associations between treatment received and treatment failure or seizure recurrence, respectively. In the Cox model, time analysis was restricted to the first 12 hours after initial control, a period when most (92%) of the seizures had recurred, and the proportional hazard assumption was not violated.

## Results

### Patient recruitment and baseline characteristics

**Figure 1:**
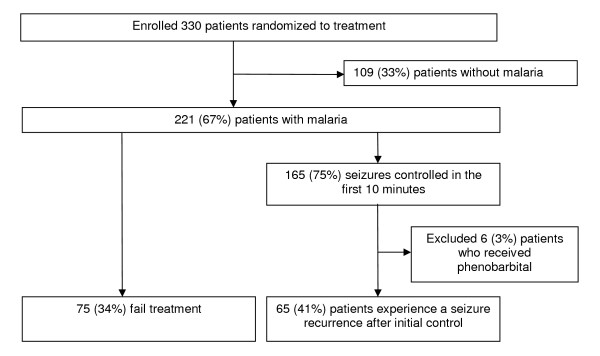
**Study profile**.

Of the 330 patients enrolled in the clinical trial, 221 (67%) had malaria and were analysed for outcomes of interest (Figure 1). Of the 221 patients with malaria, 165 (75%) had their seizure controlled within 10 minutes of receiving study medications. Of these, six (3%) patients who received phenorbarbital were excluded from the final analysis. Of the 159 evaluable study patients whose seizures were controlled in the initial 10 minutes, 65 (41%) experienced a seizure recurrence in the subsequent 24 hours. Of the 221 seizures occurring in malaria patients, 142 (64%) were associated with a fever, and 48 (22%) were observed in children diagnosed with cerebral malaria. Characteristics of seizure types in patients who had cerebral malaria and those with other forms of malaria were similar (Table 1). However, patients with cerebral malaria had greater age (p < 0.001) and were more likely to have experienced multiple seizures (p = 0.03) compared to those who had other types of malaria.

**Table 1:** Baseline characteristic of patients enrolled in the study

	**Type of malaria**	
**Characteristic**	**Cerebral malaria**	**Non-cerebral malaria**
Number	48	173
Gender (% female)	19 (40%)	90 (52%)
Number of seizures	3 (2–5.5) ^a^	2 (1–4) ^a^
Focal seizures (%)	7 (15%)	25 (14%)
Age (months)	28 (17–42)^b^	17 (12–30)^b^
Age range(months)	4–117	3–99
Blood glucose level at presentation	113 (75–172)	116 (88–155)

**NOTE**. All results are medians (IQR) unless otherwise specified. Within 24 hours before enrollment

^a ^p = 0.02

^b ^p < 0.001

### Predictors of treatment failure

In univariate analysis, the following factors were associated with an increased risk of anti-convulsant treatment failure: focal seizures, cerebral malaria, and blood glucose at presentation ≥ 200 mg/dl (11 mmol/l) (Table 2). In multivariate analysis, focal seizures, cerebral malaria, and hyperglycaemia remained as significant predictors of anti-convulsant treatment failure (Table 2). In addition, compared to midazolam, diazepam was not associated with a significant risk of treatment failure (OR for failure with diazepam 1.40; 95% CI 0.77–2.56, p = 0.26). This was in contrast to the situation in patients who did not have malaria, where diazepam was associated with a significant risk of treatment failure (56% vs. 26.5%; RR 2.11, 95% CI 1.26–3.54, p = 0.002) as we reported previously [4].

**Table 2:** Univariate and multivariate analysis for factors predicting treatment failure

		**Univariate**			**Multivariate**		
		
**Variable**	**Number of treatment failures**	**Odds ratio**	**95% CI**	**p-value**	**Odds ratio**	**95%CI**	**p-value**
**Type of seizure**							
Generalized seizure	56	-	-		1	-	-
Focal seizure	19	3.47	1.57–7.67	<0.001	3.21	1.42–7.25	0.005
**Form of disease**							
Non-cerebral malaria	52	-	-		1	-	-
Cerebral malaria	23	2.14	1.10–4.15	0.02	2.43	1.20–4.91	0.01
**Recent use of diazepam**							
No	65	-	-		^a^		
Yes	10	1.88	0.75–4.70	0.16	^a^		
**Pre-treatment blood glucose level**							
< 200 mg/dl	62	1	-		1		
≥ 200 mg/dl	13	2.85	1.16–6.95	0.01	2.84	1.11–7.20	0.02
**Age**							
> 11 months	54	1	-		1		
< 12 months	21	1.88	0.96–3.67	0.06	1.99	0.96–4.13	0.06
**Number of seizures**							
< 3	38	1	-		^a^		
≥ 3	37	1.18	0.80–2.56	0.21	^a^		
**Treatment received**							
Midazolam	37	1	-		1		
Diazepam	38	1.21	0.69–2.11	0.50	1.40	0.77–2.56	0.26
**Unconscious prior to onset of seizure**							
No	35	1	-		^a^		
Yes	37	1.60	0.90–2.85	0.11	^a^		

^a ^Not included in the final model.

**Figure 2:**
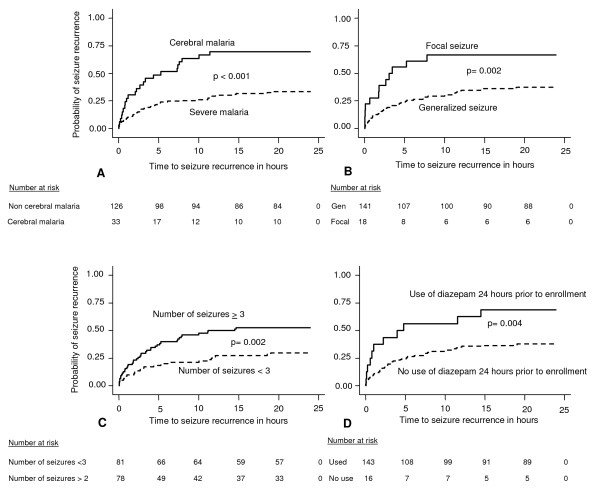
**Kaplan-Meier plots of time to first seizure recurrence within 24 hours after initial control for the subgroups indicated**. Figure 2 shows Kaplan Meier survival plots showing time to seizure recurrence within 24 hours after initial control of the seizure in the following subgroups of patients: A. Malaria type: Cerebral malaria vs. non-cerebral malaria. B. Seizure type: Focal seizures vs. generalized seizures. C. Seizure frequency *: > 2 vs. < 3. D. Prior treatment with diazepam*: Received diazepam vs. did not receive diazepam. * 24 hours prior to presentation to the acute care unit.

Predictors of seizure recurrence were also assessed using survival analysis (Figure 2). In addition to focal seizures and cerebral malaria, history of multiple seizures or prior use of diazepam was associated with a significant risk of seizure recurrence (p ≤ 0.004 for all comparisons).

In the final Cox proportional hazard model (Table 3), the following factors remained significant predictors of seizure recurrence in the subsequent 12 hours: 1) focal seizures (HR 2.86; 95%CI 1.49–5.49, p = 0.002), 2) multiple seizures (HR 2.45; 95%CI 1.42–4.23, p = 0.001, 3) cerebral malaria (HR 3.32; 95%CI 1.94–5.66, p < 0.001), 4) use of diazepam within 24 hours prior to enrollment in the study (HR 2.43 95%CI 1.19–4.95, p = 0.01), and 5) treatment with diazepam to control a seizure (HR 1.96; 95%CI 1.16–3.33, p = 0.011).

**Table 3:** Cox model for predictors of seizure recurrence within 12 hours after initial control

**Characteristic**	**Hazard ratio**	**95% CI**	**p-value**
Focal seizure	2.86	1.49–5.49	0.002
Number of seizures prior to treatment: ≥ 3	2.45	1.42–4.23	0.001
Cerebral malaria	3.32	1.94–5.66	< 0.001
Recent use of diazepam	2.43	1.19–4.95	0.01
Treatment with diazepam vs. midazolam	1.96	1.16–3.33	0.01

## Discussion

Predictors of anti-convulsant treatment failure and seizure recurrence were evaluated in Ugandan children presenting to an emergency unit with malaria complicated by prolonged seizures. Presenting with cerebral malaria, focal seizures, or hyperglycaemia were found to be independent predictors of anti-convulsant treatment failure. Predictors of seizure recurrence after initial control with an anti-convulsant included presenting with cerebral malaria, focal seizures, or multiple seizures. In addition, compared to midazolam, prior use of diazepam or initial control of a seizure with diazepam were associated with an increased hazard of experiencing a recurrence. Of note, in children with malaria, focal seizures and cerebral malaria were found to be important markers of refractory seizures, considering that they were associated with both anti-convulsant treatment failure and seizure recurrence.

In this study, children with malaria who presented with focal seizures were more likely to fail treatment than those with other seizure types. This finding is consistent with prior reports that focal seizures are a risk factor for refractory status epilepticus in adults [29]. One possible explanation for this finding is that, in contrast to generalized seizures, focal seizures may be associated with a localized structural injury representative of serious underlying brain pathology. Children presenting with cerebral malaria were also more likely to fail treatment, which is not surprising considering the severe nature of this syndrome [30]. Baseline hyperglycaemia (glucose value ≥ 200 mg/dl) (11 mmol/l) was found to be a risk factor for treatment failure. Stress hyperglycaemia, a known complication in critically ill children, has been associated with increased mortality [31-33]. It is possible that, in children with severe malaria, hyperglycaemia is a surrogate marker of ongoing serious brain disease [33]. As previously reported, considering all children with prolonged seizures, diazepam was associated with a significant risk of treatment failure compared to midazolam [4]. Considering the impact of malaria, children with prolonged seizures and malaria who received diazepam, but not those who received midazolam, were less likely to fail treatment compared to those without malaria [4]. This finding of decreased failures of diazepam in children with malaria contrasts with findings of another recent trial [34], perhaps due to differences in study design.

Risk factors for seizure recurrence in children with malaria have not been well characterized. Predictors of seizure recurrence in patients without malaria include presentation with multiple or focal seizures [35]. In this study of children with malaria, focal seizures, cerebral malaria, and presenting with multiple seizures were all independent risk factors for seizure recurrence. In addition, seizures that were controlled with diazepam were significantly more likely to recur in the subsequent 12 hours as compared to those controlled by midazolam. This finding suggests that, in the absence of alternative anti-convulsants, diazepam is likely to be administered repeatedly, exposing patients to the risk of drug induced respiratory depression [15,27], a complication that may commonly be fatal in rural sub-Saharan Africa, where assisted ventilation is generally unavailable [6,16]. In addition, diazepam induced respiratory depression may increase mortality in children with severe malaria by suppressing control of metabolic acidosis and/or increased intracranial pressure [16,13,14,17].

These predictors can be used to identify children with malaria who may benefit from alternative therapy, in the particular the use of longer-acting and safer acting anti-convulsants, such as midazolam or lorazepam. More studies are urgently needed to evaluate the effectiveness and prophylactic benefit of longer acting agents in the treatment of seizures in children with malaria.

## Conflict of interests

The authors declare that they have no competing interests. The sponsors of this study and the donors of study drugs had no role in study design, the collection, analysis, or interpretation of data or in the writing of this report.

## Authors' contributions

AM contributed to study design and coordination, collected data, supervised patient enrollment and follow-up, and analyzed and interpreted data. JB contributed to study design, supervised coordination of the study, enrollment, and clinical care of the patients and contributed to interpretation of data. GN contributed to study design and coordination, supervised enrollment and follow-up of the patients and contributed to data interpretation. SS contributed to study design, analysis and data interpretation. PJR contributed to study design and coordination, analysis and data interpretation. All authors read and approved the final manuscript.

PJR is a Doris Duke Charitable Foundation Distinguished Clinical Scientist.
